# Do tax policies discriminate against female traders? A gender framework to study informal marketplaces in Nigeria

**DOI:** 10.1002/pop4.349

**Published:** 2022-08-22

**Authors:** Imaobong Akpan, Mª Josep Cascant‐Sempere

**Affiliations:** ^1^ Design, Monitoring and Evaluation & Learning Manager Search for Common Ground Abuja Nigeria; ^2^ Development Policy & Practice Team, Faculty of Arts & Social Sciences Open University, Walton Hall Milton Keynes UK; ^3^ Department of Sociology and Social Anthropology Faculty of Social Sciences, University of Valencia Blasco Ibáñez Avenue Spain

**Keywords:** gender, informal trade, Nigeria, policymaking, taxation

## Abstract

Scholars have long debated formalizing the informal sector through taxation, but how do these processes affect gender inequalities? Our study in Nigerian markets contributes a gender framework to the equitable taxation literature on formalization. The study draws on a survey of 451 traders in 12 markets, key informant interviews, and ethnographic research in four markets of two states. We find that in at least two situations taxation policies discriminated against women implicitly: (1) male tax collectors had higher incidences of harassment on all traders and (2) taxing traders with flat taxes penalized women, as they earned less than men but were taxed the same.

## 摘要

长期以来，学者们一直在辩论通过税收将非正式部门正规化，但这些过程如何影响性别不平等？我们对尼日利亚市场的研究为关于正规化的公平税收文献提供了一个性别框架。该研究基于对12个市场的451名商人的调研、关键知情人访谈、以及在两个州四个市场中进行的民族志研究。我们发现，至少在两种情况下，税收政策暗中歧视女性:1)男性税务员对所有商人的骚扰发生率更高；2)以单一税率对商人征税会将女性置于不利地位，因为她们的收入低于男性，但征税率相同。

## Resumen

Los académicos han debatido durante mucho tiempo la formalización del sector informal a través de impuestos, pero ¿cómo afectan estos procesos a las desigualdades de género? Nuestro estudio en los mercados de Nigeria aporta un marco de género a la literatura sobre tributación equitativa sobre la formalización. El estudio se basa en una encuesta de 451 comerciantes en 12 mercados, entrevistas con informantes clave e investigación etnográfica en cuatro mercados de dos estados. Encontramos que en al menos dos situaciones, las políticas tributarias discriminaban implícitamente a las mujeres: 1) los recaudadores de impuestos masculinos tenían una mayor incidencia de acoso en todos los comerciantes; 2) gravar a los comerciantes con impuestos fijos penalizaba a las mujeres, ya que ganaban menos que los hombres pero pagaban los mismos impuestos.

## INTRODUCTION^1^

Sixty‐one percent of global employment is informal, while in Africa the percentage is 85% (ILO, 2018, pp. 13–14). Considering the large size of the economic activity not regulated or protected by the state, governments and scholars have long been interested in “formalization” or the policy processes aimed at drawing the informal sector into the tax net. Yet, research has still not shown whether these processes are egalitarian between men and women.

Many scholars have seen economic and governance opportunities in taxing informal workers, be it to access untapped revenue sources and create conditions for economic growth, or to draw the economically vulnerable into a social contract with the state. Others have questioned these optimistic versions by exposing the challenges that accompany formalization (Prichard & Boogaard, 2017; Richter, 2019). Formalization policies often move slowly, are costly, generate little revenue, face local opposition, produce unexpected results, and are no magic bullet to economic progress, positive tax–accountability linkages, and reduced marginalization.

Only more recently, scholars have studied formalization from a social justice or “equitable taxation” perspective, exploring how taxation either reduces or exacerbates inequality (Prichard, 2018). They have raised the concern that any analysis of expanding tax collection in low‐income countries must be accompanied by an exploration of the effects it creates on inequality.

However, less attention has been paid to how informal taxation affects men and women differently. According to ILO (2018, pp. 13–14), women suffer from greater vulnerability in informal employment. They also represent a higher proportion of informal employment in low and lower‐middle‐income countries. Analyzing gender inequality is vital and complements other inequality analyses of formalization processes.

This study aims to contribute to the development of a gender perspective in the debates around formalization and tax equity in the informal sector. The paper explores tax policies affecting market women in two Nigerian states. The study used a mixed‐methods approach consisting of key informant interviews, a quota‐based survey of 451 market traders^2^ in 12 local markets,^3^ and in‐depth ethnographic visits to four markets in the two Nigerian states: Southern Enugu and Northern Kaduna. Key informants interviewed included tax collectors, market union and association leaders, market traders, and academics.

The paper is structured as follows: it opens with a section on the relevance of the topic to Nigeria. It then introduces literature about formalizing informal economies from an inequality perspective, and on taxation and gender. It next suggests more conversation between this literature to better study the effects that taxing the informal sector has on gender equality. Details about the case study and methodology follow. Findings are presented in four sections analyzing gender inequalities in: Nigerian policies related to informal market taxation; face‐to‐face interactions in tax collection; and the explicit and implicit norms that structure market payments—including tax but also other tax‐like payments. The paper ends with implications for policy and theory.

## THE IMPORTANCE OF GENDER EQUALITY IN INFORMAL TAXATION TO NIGERIA

At least three factors make the topic of study relevant for Nigeria. First, the informal sector, and informal market trading, is central to Nigeria's economy. The informal sector accounts for an estimated 64% of the gross domestic product (Hoffmann & Melly, 2015) while in 2017, trade activities contributed 18% of Nigeria's GDP and delivered 14% of all employment (NBS, 2018).

Second, the trade and informal sectors are feminized. Sixty‐five percent of the 10.8 million Nigerians employed in the trade industry in 2017 were women (NBS, 2018). The National Bureau of Statistics does not disaggregate this figure—seven million women traders—into formal and informal trading. Yet, given the high proportion of women across Africa who work in the informal sector—89%, according to ILO (2018, p. 20)—it is likely that a large percentage of the roughly seven million women working in trade in Nigeria do so informally, including those working in informal local markets.

Third, women stay in the informal sector longer than men and do so in more precarious conditions. Lower levels of education, skills, and training compared to men make it harder to secure formal employment. Women are also less likely to have market knowledge and own property, making it harder to open a formal business. Finally, women are more likely to have family and community commitments, limiting the attention according to their careers (Carroll, 2011; Grown & Valodia, 2010; Williams, 2009).

## AN INEQUALITY APPROACH TO TAXING THE INFORMAL SECTOR

The inequality or equitable taxation approach explores how taxes contribute to reducing or expanding inequality. Scholars of this approach claim that the interest in expanding tax collection in low‐income countries needs to come hand in hand with an analysis of how that revenue is raised and how it affects the poor (Prichard, 2018; Prichard & Boogaard, 2017).

At the heart of this approach lay concepts such as progressive taxation systems, which impose higher taxes to those with higher incomes, and that are credited for helping reduce inequality. Conversely, regressive taxation imposes a larger relative burden on low‐income earners and is thought to widen inequality.

The picture however is more complex, as tax equity depends not only on how revenue is collected but also on how it is spent. For instance, some have argued that states may not translate revenues—whatever their nature—into public benefits, thus reinforcing inequalities and creating skepticism among taxpayers. Conversely, regressive taxation can contribute to equality if it is spent to benefit poorer groups (e.g., Kato & Tanaka, 2018).

The same complex picture is found in studies exploring the informal sector. For instance, Dube and Casale (2016) found deepening inequalities with the introduction of presumptive taxation in Zimbabwe. “Presumptive taxation” is a flat, regressive tax that is characteristic of the informal sector in low‐income countries (Lahey, 2018, p. 39). Tax authorities assess earners of income in the informal sector—be they self‐employed individuals or larger businesses—based on estimated income and set a single flat tax for all, regardless of the real income earned. In a more recent study, van den Boogaard et al. (2019) differentiate between statutory taxes such as presumptive taxation and other tax‐like payments and found taxpayer perceptions in Sierra Leone to be more positive towards tax‐like payments. Taxpayers, the authors suggest, were more likely to believe that the actor levying these payments would use them to deliver community benefits. This is partly corroborated by another study highlighting the mistrust that women traders had of customs officials and their tendency to demand more money than the official tax rate to satisfy their personal benefit (Yusuff, 2014, p. 26).

Two implications emanate from this growing literature. First, both tax collection and tax usage (how tax is spent) interplay in affecting inequality. Second, inequalities derive not only from statutory taxes but also from other informal tax‐like payments. Tax‐like payments include fees, levies, and rates raised in cash, in kind, or in labor outside the legal tax system by both state and non‐state actors (van den Boogaard, 2018; van den Boogaard & Prichard, 2016).

Bringing these types of payments together recognizes the real total amount borne by citizens, which in turn makes visible all the spectrum of local actors involved in collecting (and spending) revenue and their role in local development. In some cases, tax‐like payments can be higher and less transparent than tax payments (Siebert & Mbise, 2018; van den Boogaard, 2018). In other cases, they can represent a substantial source of local and central government revenue (Deng & Smyth, 2000) and be decisive in the provision of redistributive mechanisms such as public services, cash transfers, and subsidies. Van den Boogaard and Prichard (2016) suggest that all payments be analyzed instead of a more conventional analysis of tax payments only.

A third insight from the equitable tax literature is that it does not always consider gender. This paper contributes to growing gender analysis in the inequality approach to the formalization debate, and suggests that much of the growing literature on gender and taxation has been doing exactly that—looking at gender inequalities in taxation.

## A GENDER FRAMEWORK TO STUDY INEQUALITY IN INFORMAL TAXATION

Scholars of the gender and taxation literature have looked at inequalities from different perspectives, often focusing more on the formal than the informal sector. To date, the gendered effects of informal taxation continue to be little studied, even though taxing the informal sector is becoming a priority for many countries (Lahey, 2018, p. 39). This review focuses on the research that studies both formal and informal taxation, or that has specifically focused on the informal sector. This includes explicit and implicit discrimination, the differentiated effects of presumptive taxation and tax‐like payments on men and women, and tax harassment.

In terms of social norms in informal tax collection contexts, several researchers have expressed concerns about implicit tax biases that contribute to inequalities between men and women (Barnett & Grown, 2004; Carroll, 2011; Grown & Valodia, 2010; Stotsky, 1996). While explicit tax biases result from specific provisions of the law that deliberately treat men and women differently, implicit biases occur when tax structures appear to treat men and women equally, but those structures have an unequal impact in practice. This distinction draws on the legal concepts of direct and indirect discrimination stipulated in national and international law, such as the Committee on the Elimination of Discrimination Against Women (CEDAW). Direct sex discrimination is generally defined as less favorable treatment with an explicit distinction between different sexes. Indirect discrimination refers to apparently neutral provisions or practices which might result in a disadvantage for one sex or another due to existing socioeconomic and cultural differences.

In her seminal work, Stotsky (1996) identifies several types of tax in which explicit and implicit gender biases could exist, namely personal income tax, corporate income tax, commodity tax (e.g., VAT, selective taxes), and trade taxes. However, specific taxes charged in the informal sector such as presumptive taxation or tax‐like payments are not mentioned. Other scholars (Casale, 2012; Wanjala & Were, 2011) have studied the relationship between VAT and gender equity in African contexts, including Kenya and South Africa. While VAT and presumptive taxation tax different aspects of an economy (consumption vs. income), they are both flat, regressive taxes. Thus, they could create similarly gendered effects.

A study in Ghana indicates that women in the informal sector who earn more money pay a lower percentage of their monthly income in tax (8%) compared to a higher percentage (37%) paid by women who earn less per month (Carroll, 2011). This variation also applies to male and female traders, with men paying relatively less than women, an aspect that has been evidenced for Zimbabwe (Ligomeka, 2019) and that is further explored in this study for Nigeria. In terms of tax‐like payments, van den Boogaard (2018) found that women in Sierra Leone are more likely to pay informal taxes, or payments to access local public goods and services, while men are more likely to pay formal taxes.

In terms of gendered interactions during informal tax collection processes, most studies have studied the relationship between tax collectors and market vendors (Carroll, 2011; Ligomeka, 2019; Siebert & Mbise, 2018; van den Boogaard & Prichard, 2016). The evidence is mixed. Some studies report difficult relationships, including harassment (especially of foreign and illiterate women), seizure of possessions, bribes, and coerced sexual favors. Other studies evidence mutual respect between women traders and tax collectors, with tax exemptions being common for elderly women, the sick, young mothers, and widows.

Less has been written on the policy implications deriving from those cases where tax harassment has been evidenced, such as sanctions and job dismissals for perpetrators. Mwondha et al. (2018) call for the recruitment of more women into the Uganda Revenue Authority. However, this call is based on evidence showing women's higher job performance and is not related to issues of tax harassment.

Singularly, van den Boogaard (2018) studies how differentiated payments of tax—men paying more formal taxes and women paying more informal taxes—affect interactions with government and chiefdom officials, relationships within households, and roles of political representation associated with paying formal taxes.

This study merges the equitable taxation literature with the gender and taxation literature to study the Nigerian case. This theoretical integration helps bring a broader analysis of gender inequalities in the taxation of the informal sector, including diverse market payments (both statutory and nonstatutory), human interactions between government tax collectors and traders, and explicit and implicit policy biases.

## METHODOLOGY

The states of Enugu and Kaduna were selected to explore the interaction between gender and taxation as they represent different cultural and religious contexts. Enugu State is in southeast Nigeria, is mostly of Igbo culture and language, and is majority Christian. Kaduna State is in the northwest and is predominantly Hausa and mixed faith—northern Kaduna is mostly Muslim and southern Kaduna mostly Christian.^4^ Both northern and southern Kaduna were surveyed (see Table 1). The choice was also practical, as the research partner Christian Aid Nigeria, an NGO, had a strong presence in both states, which facilitated fieldwork.

**Table 1:** Formal and informal markets selected for data collection by location

State	Senatorial district	Local government area	Community	Markets visited	
				Formal	Informal
Enugu	Enugu East	Nkanu West	Akpugo	Oriemba	‐
			Agbani	‐	Eke Agbani
	Enugu North	Nsukka	Nsukka	Ogige	‐
			Edeoballa	‐	Eke Ohuru
	Enugu West	Udi	Ngwo	Aria (New Market)*	Eke Ngwo*
Kaduna	Kaduna Central	Kaduna City	Kaduna	Television (Yam Market)*	Kakuri Meat Market*
	Kaduna North	Zaria	Zaria	Tudun Wada	Gwarqwaji
	Kaduna South	Kafanchan	Kafanchan	Kafanchan Main market	Yakowa

*Source*: authors’ elaboration.

* Indicates ethnographic work.

Creating the sampling strategy was challenging, as no official data exists on the total number of markets (either formal or informal) in Enugu and Kaduna, or for Nigeria.^5^ To overcome this lack of data, we consulted local markets located on Google Maps and relied on local informants to identify a total of 47 markets, 28 in Enugu State and 19 in Kaduna State. These were clearly too few and not the full number of markets needed to cover the large population of the two states. Thus, we could not find the total number of markets—the initial population of markets for our sampling—with this method.

Even more problematic than identifying markets was finding the total number of local market traders, let alone disaggregated by gender. The most approximate data found was the number of registered micro‐enterprises by state, and the number of men and women employed by these (SMEDAN & NBS, 2013). However, these data likely miss many unregistered people, including the market traders that are the population of interest in this study. Also, data on registered micro‐enterprises had not been updated since 2013.

Accounting for these constraints, the study relied on a nonrepresentative geographical quota system for sampling. A local government area was selected from each of the three senatorial districts that make up each state. For each senatorial district, the local government area containing the main city was selected, as main cities were likely to provide the most developed gender and taxation policies, as well as accessible data in terms of policy and practice. Within each local government area, the most populated markets (both formal and informal) were selected.

This study commenced with a review of documents and key informant interviews in the capital city of Abuja. Findings from the review then informed the development of field tools used for key informant interviews, the survey, and the ethnographic visits to the markets.

Each study method addressed different aspects of the research questions. Secondary data and key informant interviews were used to examine the legal and policy aspects of gender and taxation in the marketplace. The survey and ethnographic work were used to explore the ways that traders experience tax collection in the markets. We spoke to both market leaders and market traders to compare views.

Two stages of key informant interviews were conducted. The first stage explored national aspects of market taxation with a representative from the Federal Inland Revenue Service and a representative of the Nigerian Tax Research Network. The second stage was conducted in Enugu and Kaduna States with 26 experts (15 in Enugu and 11 in Kaduna) in tax, trade and gender from academia, civil society, government and market leadership. In total, 28 key informant interviews were conducted (see Table 2).

**Table 2:** Demographics of key informant interviewees by location

Organisation type represented	National	Enugu State	Kaduna State
Civil society	1 (male)	1 (male)	1 (female)
Tax authority	1 (male)	1 (male)	1 (male)
Local government	0	1 (female)	1 (male)
Academia	0	1 (male)	0
Market union leadership	0	7 (3 male, 4 female)	6 (3 male, 3 female)
Market tax collectors	0	4 (2 male, 2 female)	2 (male)
Total	2 (2 male)	15 (8 male, 7 female)	11 (7 male, 4 female)

*Source*: authors’ elaboration.

Additionally, 451 survey questionnaires were collected in 12 markets. Two hundred and twelve respondents (47%) were female, and 239 respondents (53%) were male. In each market, we collected an average of 37 questionnaires to cover a variety of traders per type of product (e.g., vegetables, fruits, fish, meat, clothes) and gender (men and women). There was one respondent per questionnaire.

Ethnographic studies were limited to four of the 12 markets visited. The markets chosen were the largest and most popular formal and informal markets in one selected senatorial district per state (see Table 1 marked with an asterisk). Ethnographic work gave researchers more time for market observation and for more flexible interviews with market leaders and market traders. Its collection ended when we saturated all the visible market profiles by product sold and by gender. Interviews were not fully transcribed from the recordings due to budget and time constraints. However, thorough interview summaries were written, and care was taken to ensure that the quotes used were identical to what was recorded.

## POLICIES ON INFORMAL TAXATION ARE GENDER‐BLIND IN NIGERIA

Nigeria operates a decentralized tax system with three tiers of government levels—federal, state, and local—that work autonomously in administering their taxes. The country generates revenue through a pool of taxes such as Personal Income Tax, Corporate Tax, Petroleum Profits Tax, Value‐Added Tax, and Tariffs. Nigeria's tax compliance rate is relatively low compared to other countries.^6^ This could be due to Nigeria's weak revenue administration capacity and a lack of data on the country's large informal sector.

National tax policies, and Enugu and Kaduna state tax laws,^7^ are gender‐blind regarding the informal sector and local markets. Existing policies and laws are not explicitly biased for or against any gender. No specific tax concessions are mentioned, nor specific goods or services taxed differently because of the fact that they are primarily sold to or provided by men or women. As seen below, this situation discriminates against women in several ways.

The National Tax Policy allows for different types of taxes based on one's income bracket and income‐generating activities, including personal income tax, value added tax, and (in the informal sector) presumptive tax. However, these distinctions make no reference to different forms or rates of taxation for men and women (Federal Ministry of Finance, 2016).

The Enugu State Tax Law specifies a flat presumptive tax of N1,000 (US$2.80) per store (ESBIR & SPARC, 2014). The Enugu State Government Board of Internal Revenue (ESBIR) has made efforts to partner with the leaders of market associations and transport unions to collect taxes. However, no assessment guides the current rates. Some Nigerian civil society associations have reported that methods of tax collection are contracted out, which may be resulting in the over‐taxation of small businesses (CISLAC & JDPC, 2014).

The Kaduna State Tax Law specifies a flat presumptive tax of N2,500 (US$6.90) for microbusinesses in the informal sector (Kaduna State Government, 2016). The state has specific tax rates for slaughter stock per head. However, the law does not explain why slaughter stock is cited, nor if this is in addition to the flat tax of N2,500. While not specified in law, the owners of slaughter stock in Kaduna State, as in other Nigerian states, are predominantly men.

The above evidence shows that flat presumptive taxes differ between states—N1,000 in Enugu and N2,500 in Kaduna—and that some special provisions are made for certain products in Kaduna, but not in Enugu. Except for slaughter stock, presumptive taxation is not subdivided according to different expected incomes, for instance, based on the types of products sold. Current state tax laws do not include recommendations on gender equity in the taxation of the informal sector.

To date, neither the national policy nor the state policies adopt a position around the identification and prevention of harassment in tax collection or the differentiated effects of other tax‐like payments on men and women, besides presumptive taxation.

## GENDER BIASES IN TAX COLLECTION INTERACTIONS: MALE TAX COLLECTORS HARASS TRADERS MORE THAN FEMALE TAX COLLECTORS

Three types of market actors were involved in the process of tax collection for all of the markets visited, both formal and informal. These were frontline government workers, market unions, and market associations. This study focuses only on frontline government tax collectors.^8^

To explore the existing relationship between market vendors and tax collectors, researchers asked vendors about the occurrence of negative incidents in the process of tax collection. Table 3 shows the total number of incidents reported by market vendors, disaggregated by gender, since they began trading in their current market. Similar numbers were reported by female and male vendors for increases to the tax amount, confiscation of goods, physical and verbal harassment, demand for bribes, and unofficial reductions to the tax amount. The only significant gender difference found among vendors was the three incidents of demands for sexual favors, which were reported only by female vendors. One incident of sexual harassment was reported in the survey by a middle‐aged woman in the informal Eke Agbani Market in Enugu State, and two were reported by young female traders in Kaduna State, in the formal Television Market and the informal Yakowa Market.

**Table 3:** Number of negative incidents reported by market vendors by gender

Type of incident reported	Number of incidents reported by all vendors	Number of incidents reported by female vendors	Number of incidents reported by male vendors	% of all incidents reported by all vendors
Increased tax amount	105	47	58	23.3%
Physical and verbal harassment	98	43	55	21.7%
Confiscation of goods	80	37	43	17.7%
Demand for bribes	17	9	8	3.8%
Reduced tax amount	12	6	6	2.7%
Demand for sexual favours	3	3	0	0.7%
Total traders reporting	315	145	170	69.85%
Total traders not reporting	136	67	69	30.15%
Total market traders	451	212	239	100%

*Source*: Survey conducted among market men and women in Enugu and Kaduna States, 2018.

While reports of sexual harassment are low compared to other reported incidents (0.7% of all reported incidents), it is likely that this type of incident was underreported in the survey due to its sensitive nature. For instance, the chairman of Aria Market in Enugu reported observing cases of sexual harassment, but no interviewed vendor in the same market reported the issue. The chairman noted in his interview:

Women behave [in a certain way] and say the men have come asking them to befriend them and we have heard of one case before. So, when we meet such women, we send a female taskforce person to them.

All reported demands for sexual favors were perpetrated by male tax collectors (see Table 4). Additionally, male tax collectors were responsible for 97.9% of reported cases of physical and verbal harassment, 91.6% of reported cases of confiscation of goods, and 83.3% of reported cases of unofficial reduced tax amounts. Overall, 81.6% of all reported incidents were committed by male tax collectors despite the fact that male tax collectors make up only 73% of all tax collectors in the markets surveyed.

**Table 4:** Percentages of negative incidents reported by market vendors by tax collector gender

Type of reported incident	% of total incidents	% of incidents by female tax collectors	% of incidents by male tax collectors
Increased tax amount	23.3%	43.7%	56.3%
Physical and verbal harassment	21.7%	2.1%	97.9%
Confiscation of goods	17.7%	8.4%	91.6%
Demand for bribes	3.8%	41.5%	58.5%
Reduced tax amount	2.7%	16.7%	83.3%
Demand for sexual favors	0.7%	0.0%	100%
Nonresponses	30.15%	n/a	n/a
Total	100%	18.4%	81.6%

*Source*: Survey conducted among market men and women in Enugu and Kaduna States, 2018.

The incident of reduced tax amounts can be read in two ways: it is either a reflection of a positive understanding by tax collectors about the hardships faced by some traders, or it is a tactic used by tax collectors to extract favors from the traders. The findings of this study do not shed light on which reading is more accurate although the second reading is more likely given that market traders considered these tax reductions as negative. Another option for this negative reading is that traders want to pay what is due to avoid problems, no more and no less.

Interestingly, incidents of increasing tax amounts and asking for bribes were reported equally for female and male tax collectors. Keeping in mind that female tax collectors represent only 27% of all tax collectors in the markets visited, the reported incidents of increasing tax amounts and requesting bribes are surprisingly high for female tax collectors. The main ways that female tax collectors were reported to treat market vendors negatively is thus by increasing their tax amount (43.7% of all incidents among female collectors) and demanding bribes (41.5% of all incidents among female collectors). Overall, however, female tax collectors were responsible for only 18.4% of all the negative incidents reported, which is less than would be expected given their representation among the total number of tax collectors.

Market association leaders interviewed in both states noted that the confiscation of goods was a strategy tax collectors used when vendors had failed to pay their taxes within a stipulated period and following repeated warnings from tax collectors. Vendors reported that only 8.4% of incidents of confiscated goods were carried out by female tax collectors, compared to 91.6% by men (see Table 4). Additionally, female tax collectors involved in the confiscation of goods reportedly did so in nonviolent ways. A market association leader in Ogige Market, Enugu State, attributed this tendency to their calmer temperaments and lower physical strength. The association leader specifically mentioned that female tax collectors did not fight vendors. Others agreed:

Some [vendors] will say the amount is too much. Some will not even talk to you. They go and pay and when they come [to collect the goods confiscated], [it] depends on [the] interaction, [on] how I approach the person he will still go and pay. We don't fight. (Female tax collector, Ogige Market, Enugu State)

Male tax collectors, in contrast, were reported to be more direct when confiscating goods:

They [market vendors] complain sometimes… especially those that don't have money immediately because they [male tax collectors] will collect it by force. (Secretary, Aria Market Union, Enugu State)

If the chairman of the market should find it difficult collecting money, they will call us [male tax collectors]. Once we ask the person why and he/she gives no reasonable reason, we lock up the person's store at night. Then [there] is a peace committee who will now settle the issue. (Male tax collector, Ogige Market, Enugu State)

Leveraging the strengths of female tax collectors, a female tax collector in Enugu State noted the existence of tax collection teams composed of both females and males. Mixed‐gender teams were observed in the formal Aria and Ogige Markets in Enugu State during the visits. This strategy was also confirmed by a male market union leader in the formal market of Kafanchan Central, Kaduna, who stated:

They come in groups to the market and the male collectors are usually more than the female. In a group of ten, two out of ten represent female collectors. For instance, in my own shop when they arrive, their attitude is mild. They ask questions like have you paid, show me the receipt or evidence of payment.

These examples reveal the merit of having more women in tax collection roles. They also demonstrate cases in which steps have been taken to integrate female tax collectors in the tax collection teams. Steps include the intentional creation of mixed female and male tax collection teams, and female tax collectors replacing male tax collectors for cases of reported sexual harassment by male tax collectors.

## NO EXPLICIT GENDER BIAS IN MARKET PAYMENTS: FEMALE AND MALE TRADERS PAY THE SAME AMOUNT OF TAX AND TAX‐LIKE PAYMENTS

No explicit gender discrimination was found in terms of tax and tax‐like payments in the markets visited—with the exception of rents, men and women paid exactly the same amounts of money for each of the payments. Sixty‐nine percent of women reported that they paid the same tax as men, and 70% of men reported that they paid the same tax as women. Thirty‐one percent of women and 30% of men surveyed did not know if women paid more tax than men.

However, traders—both female and male—were found to be paying higher presumptive taxes than what is stipulated in their respective state laws in 9 of the 12 markets visited. This tax payment increase was in effect for all of the markets visited in Enugu State, and for some of the markets in Kaduna State. Concern regarding the higher tax rates paid by traders has also been highlighted in a study from Anambra State, a neighboring state to Enugu (CISLAC & JDPC, 2014). This has the effect of exacerbating implicit gender biases, as suggested below.

For all surveyed markets, annual presumptive taxes range from N1,000 (US$2.80) to N12,000 (US$33.30), depending on the location and size of the shop. In Enugu state, market taxes range from N1,200 (US$3.30) in the formal Aria Market to N3,500 (US$9.70) in the formal Oriemba Market. These figures can be compared to the presumptive tax rate of N1,000 (US$2.80) stipulated in the Enugu State Tax Law. Only one female trader in the formal Aria Market mentioned paying the stipulated tax rate.

Similarly, the stipulated presumptive tax rate in the Kaduna State Tax Law is N2,500 (US$6.90).^9^ In practice, however, Kaduna State has a bracket of tax rates ranging from N1,000 (US$2.80) in the informal Kakuri Market, to N10,000 ($27.80) in the main Kafanchan Market and N12,000 ($33.30) in Tudun Wada Market, which are both formal markets. In Kaduna State, vendors in some markets are taxed at a lower rate, including the formal Television Market and the informal Kakuri Meat Market, and Gwarqwaji Market, in which taxes range from N1,000 (US$2.80) to N2,400 (US$6.70). However, the other three markets visited in Kaduna State—Tudun Wada, Kafanchan Main Market, and Yakowa—are also taxed at a higher rate than stipulated by law, with the real tax rate ranging from N3,000 (US$8.30) to N12,000 (US$33.30).

Aside from presumptive taxes, vendors pay market fees and other tax‐like payments (see Table 5). The literature on informal taxation recommends the inclusion of both tax and tax‐like payments, as together they represent the true financial cost of doing business and the true picture of potential gender inequalities.

**Table 5:** Tax‐like payments in the markets visited, formal and informal

Type of fee	Description	Cost	Frequency
Hawkers’ licence fees	Cost paid by hawkers each day they trade in the market	N10–N100 (US$0.03–US$0.30)	Daily
Toilet fees	Amount paid by market vendors for use of toilets within the market	N20–N50 (US$0.05–US$0.13)	Per use
Water fees	Costs paid for collection of water from water sources within the market	From N20 (US$0.05) per container	Per use, depending on size
Security fees	Fees contributed for payment of security personnel employed to guard the market	N200 (US$0.55)	Monthly
Sanitation fees	Cost paid by market vendors for personnel who collect and dispose of refuse generated within the market	N200–N500 (US$0.55–US$1.39)	n/a
Electricity fees	Cost paid for using electricity in the market, based on individual or joint shop metered readings, or estimations by electricity service providers. Only some markets have this service.	n/a	As per meter reading or estimation
Market or shop rents	Payments for use of market stalls, either to the government or to private landlords. Small rents are for those paying for ground space in front of others’ shops.	N50–N150,000 (US$0.13–US$416)	Annually
Market access charges	Fees paid by vendors to bring their goods into the market, charged per bag or basket depending on the type of product, e.g. rice, or per trailer or lorry load, depending on the amount of goods brought in.	N20 (US$0.05) for products sold in bags, N5,000 (US$13.90) for trucks with goods	Per bag/per delivery

*Source*: Surveys conducted among market traders in Enugu and Kaduna States, 2018. Electricity and market access charge data were collected from interviews with market union leaders.

**Figure 1:**
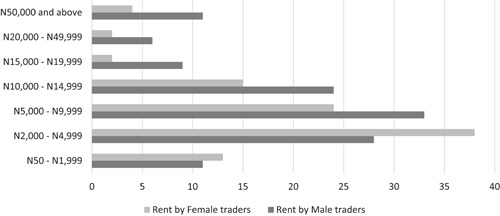
Annual rents paid by female and male market traders, in Naira. *Source*: Survey conducted among market traders, Enugu and Kaduna States, 2018.

All of these tax‐like payments were paid equally by men and women traders in the markets surveyed. The only exception was shop rents, according to market association leaders interviewed in Enugu and Kaduna States. While rent is essentially the same for all vendors regardless of gender, the fact that male vendors are able to afford more strategic locations closer to market entrances, and larger shop sizes, implies that they pay more rent accordingly. Figure 1 shows that, in the markets visited, more female traders rent shops priced between N50 and N4,999 (US$0.10–US$13.90) annually, while more male traders rent those priced at N5,000 (US$13.90) and above.

## IMPLICIT GENDER BIAS IN MARKET PAYMENTS: WOMEN TRADERS EARN LESS THAN MEN BUT PAY THE SAME PRESUMPTIVE TAX AND TAX‐LIKE PAYMENTS

Market traders complain about presumptive taxation, noting that the same amount of tax is paid by traders with different earnings. This situation is proportionally unfair to those earning less for their trade. A male market union leader in Enugu state illustrated the situation:

Somebody can have a shop where the goods are worth more than N10 million [US$28,000] and he will pay just N1,000 [US$2.80]. The neighbor can have products that are less than N200,000 [US$554], but they pay the same amount [of tax]. So, there is no equality in taxing there.

This uneven relationship between earnings and tax also has a gender dimension, as women tend to earn less than their male counterparts. As a result, it is perhaps unsurprising that women traders made up 60% of the 76% of surveyed market vendors who felt that taxes are too high.

To analyze the earnings‐tax relationship in more detail, it helps to look at the gender differences in the types of goods and services sold. These were similar in all of the markets visited, whether formal or informal. However, there were differences between states in the goods and services sold by either men or women (see Table 6).

**Table 6:** Goods and services sold in the markets visited by gender

Goods and services	Gender of sellers in Enugu State	Gender of sellers in Kaduna State
Barbing services	Only men	Only men
Cobbling	Only men	Only men
Building materials	Mostly men	Only men
Electricals/Electronics	Mostly men	Only men
Meat	Both men and women	Only men
Provisions	Both men and women	Mostly men
Fish	Both men and women	Both men and women
Clothing, shoes and bags	Both men and women	Mostly women
Sale of cooked food and drinks	Both men and women	Only women
Fruits	Mostly women	Mostly men
Food stuffs	Mostly women	Both men and women
Vegetables	Mostly women	Mostly women
Hairdressing services	Mostly women	Only women

*Source*: Market ethnography and survey among market traders, Enugu and Kaduna States, 2018.

Economic factors, which are often unequally distributed along gender lines, influence the types of goods and services being sold. For instance, high‐worth goods such as building materials and electronics are mostly sold by men who have the available capital to invest in inventory.

Similarly, more male traders were found in wholesale markets such as the formal Tudun Wada Market in Kaduna North; only 6% of survey respondents from this market were female. Low representation of female traders could be attributed to the market being located in Zaria City in Kaduna North, a predominantly Muslim area, where cultural expectations lead men to be those working in trading. However, the lower number of female traders in Tudun Wada Market is also likely due to the high capital requirements for wholesale trading.^10^ Consistent with the argument that it is capital costs, more than culture, that keeps female traders out of some markets, the informal Gwarqwaji Market, located in the same district and vicinity, has a much higher percentage of female traders (23%) than does the Tudun Wada Market. However, this percentage of women traders is still small in comparison to the other markets surveyed. Although similar goods are sold in Gwarqwaji Market, they are sold in retail quantities. For instance, rice, beans, and maize are mostly sold in large bags in Tudun Wada Market, but in cup measurements in Gwarqwaji Market. The lower capital requirements to participate in retail‐quantity trade allows a higher percentage of women to operate in the market.

The formal wholesale markets included in this study also tended to have higher presumptive taxes than the informal markets where more women participated in trading. Tax in the informal Gwarqwaji Market, for instance, is N2,000 (US$5.50). This rate is N10,000 (US$27.80) lower than the N12,000 (US$33.30) charged in Tudun Wada Market, where there are fewer women.

This dynamic of formal markets featuring higher taxes and lower representation of female traders was also found in the districts of Kaduna South and Kaduna Central. In the main formal market of Kafanchan, Kaduna South, the presumptive tax is as high as N10,000 (US$27.80) and women represent 39% of survey respondents. These results compare to the informal market of Yakowa, in the same vicinity, that has a tax rate of N5,000 (US$13.90) and 50% female traders. Similarly, in Kaduna Central, the formal Television Market has a higher tax rate of N2,400 (US$6.70) and fewer women traders (64.3%) than the informal Kakuri Market, with a tax rate of N1,500 (US$4.20) and 72.3% female traders. This dynamic indicates a trend in Kaduna State that connects informal markets, lower taxes, and more women traders. Such a distinct pattern was not found in Enugu State, where the percentages of women traders in formal and informal markets were similar.

**Figure 2:**
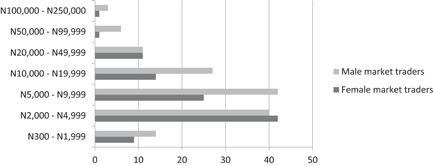
Estimated average weekly earnings of female and male market traders, in Naira. *Source*: Survey conducted among market men and women in Enugu and Kaduna States, 2018.

The results presented above, in which higher tax rates are more prevalent in markets with more male traders, might lead one to think that male traders pay more taxes than female traders. However, a comparison of the revenue generated by men and women from all visited markets does not support this view. Figure 2 shows the estimated average weekly earnings of men and women in all of the visited markets. The number of women traders in each category noticeably decreases as earnings increase.

**Figure 3:**
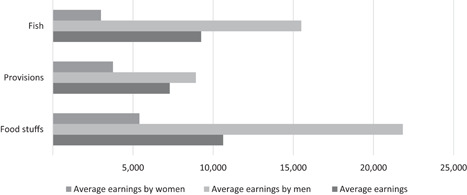
Estimated average weekly earnings of female and male traders by selected product, in Naira. *Source*: Survey conducted among market men and women in Enugu and Kaduna States, 2018.

In addition to this analysis, the average weekly earnings for a selection of items sold by both men and women are compared (see Table 6). The selected items include fish, foodstuffs (staples like rice, garri, beans, and oil, but not fish or meat) and provisions (cereals, milk, oats, and spices and bathroom supplies like toilet rolls and detergent). For these items, average earnings by women vendors are consistently lower than the average earnings for all vendors selling the same product (see Figure 3). Moreover, male vendors consistently have considerably higher average earnings on all products, whether those products are sold by both men and women (as with fish in Enugu and Kaduna), mostly by men (as with provisions in Kaduna State), or mostly by women (as with foodstuffs in Enugu State).

For fish, a product sold by both men and women, the average weekly earnings of all fish sellers were reported to be N9,250 (US$26.40). However, the average weekly earnings for female fish sellers were just N3,000 (US$8.30), less than a third of the overall average. In contrast, the average weekly earnings of male fish sellers were reported to be almost double the average for all fish sellers at N15,500 (US$43).

Similar trends were found for the other two products analyzed. With provisions, the average earnings for all traders were N7,289 (US$20.20), the average earnings for female provision traders were N3,750 (US$10.40) and the average earnings for male provision traders was N8,923 (US$24.80). With foodstuffs, the average earnings for female sellers were reported to be N5,400 (US$15), almost half of the N10,626 (US$29.50) average earnings for all foodstuff traders and about a quarter of the average earnings for male foodstuff traders at N21,833 (US$60.60).

Considering that taxation of market traders in Nigeria is presumptive across all markets visited, the lower average earnings of women compared to men introduces an implicit tax bias against women. Thus, while it seems that men pay the same or even more taxes than women, looking at the amount of taxes paid relative to earnings shows that women pay considerably more.

Furthermore, as seen in the previous section, in most markets (both formal and informal) traders are charged higher taxes than the rate stipulated in the relevant state tax law. While this increase is borne equally by men and women, it may affect women more severely, as they are already in a more precarious financial position. Added to these increases is the fact that unpaid care work^11^ keeps women from earning more revenue, thus sustaining the negative effects of implicit tax biases on women.

Market association leaders in both states noted that, in some cases, child and homecare responsibilities informed women's choices about the types of goods and services they provide, the amount of time they spend in the market, and by extension their earnings. This observation is supported by the 2017 labor force statistics report produced by the National Bureau of Statistics, which points that male traders working more than 40 h per week spend 17.1% more time in trading than female traders in the same time category of more than 40 h per week (NBS, 2018).

The same implicit gender bias rationale observed for the payment of presumptive tax applies to the series of tax‐like payments demonstrated in Table 5. The case of toilet fees could be aggravated by the fact that women use toilets more (Siebert & Mbise, 2018). Women need more privacy when using a toilet, as well as more frequent use at times during their menstrual cycle. Indirectly, this requirement introduces yet another discrimination against female traders because toilet fees exist at a flat rate for all vendors in the market, yet women earn less *and* use the toilets more.

However, surveyed traders, both men and women, did not spontaneously raise concerns about issues around limited toilet access and toilet fees as meaningfully affecting their trade. Only one female dressmaker in the formal Television Market, Kaduna State, mentioned the lack of toilets in the market despite the fee paid. She stated that the private toilets are not well kept, and that she has to leave the market to a neighboring house each time she needs to use the toilet. This inconvenience likely means, in turn, some potential loss in earnings during her absence from her business establishment.

## CONCLUSIONS

Formalization processes of taxing informal economies can be unequal for men and women in different ways, as seen in the case of Nigeria.

Two primary findings emerged from this study: (1) using male tax collectors in markets increased harassment and violence in tax collection from traders and (2) the implementation of a flat presumptive tax, in addition to other flat market payments, introduced a negative bias towards women traders, because of their lower earnings. Informal taxation policies inadvertently discriminated against businesswomen, as they did not explicitly address these two issues.

Regarding the first finding, policymakers and tax authorities should ensure a larger presence of female tax collectors in markets to reduce incidents of physical, verbal, and sexual harassment. Some of the markets studied have created mixed‐gender tax collection groups and replaced male tax collectors reported for sexually harassing women with female tax collectors. Future policy endeavors should scale up these initiatives. Including more female workers in the local tax force should come together with gender training for all tax collectors on all types of harassment, as well as zero tolerance policies and specific sanctions for perpetrators of sexual harassment.

Regarding the second finding, policymakers and tax authorities should include a segmented system of presumptive taxation based on actual earnings, or at least based on the type of product sold.^12^ Such segmentation would help alleviate the impact of implicit tax bias inherent in the current presumptive taxation system on female market traders, as well as other traders with low earnings. The national tax policy allows for different types of taxes based on one's income bracket and income‐generating activities. These aspects of the federal system should be incorporated into state tax laws. Additionally, any policy measures related to monitoring unofficial tax increases, and to supporting conciliation between unpaid care work and paid work, will only improve the hidden factors that make a supposedly neutral tax rate unfair to female traders in practice.

Another key element to the area of gender equality in informal taxation is the integration of tax‐like payments in policy and analysis as they affect traders' pockets as much as tax payments do—sometimes even more. As observed, presumptive taxation—taxes defined by statutory laws—were N1,000 in Enugu and N2,500 in Kaduna per year, while some tax‐like payments—such as security fees—were N200 per month, thus N2,400 per year, or as high as N150,000 per year for renting the best shop spaces.

The higher taxes that traders reported paying in 9 of the 12 markets visited, compared to what is stipulated in state laws, is another example of payments that stay outside statutory laws. While in Enugu all the figures charged were higher than the legal amount—a range of N1,200–N3,500 for a stipulated amount of N1,000– the Kaduna figures were larger and smaller, with a range of N1,000–N12,000 for a stipulated amount of N2,500. Bribes to tax collectors could explain the increases. Ad‐hoc exemptions or concessions made by tax collectors could explain the decreases. Tax authorities should monitor increases as they affect female entrepreneurship, given their regressive nature and women's lower income.

In particular, toilet fees are a tax‐like payment that adds an additional strain on female traders, as they need the safety and privacy of the toilet more than men for their physical and menstrual‐related needs, as noted by Siebert and Mbise (2018). Female traders end up wasting working time going outside markets for their needs or, alternatively, paying more than men for the toilet service in the market where they work.

Equitable taxation inquiry should accompany any research and policymaking on formalization processes, and gender equality cannot be outside that endeavor. Expanding the tax net should come with an analysis of the (gender) inequalities it creates. Including tax‐like payments within a broader definition of informal taxation and analyzing the impact of implicit gender biases in the informal sector should better guarantee that informal tax policies help reduce, rather than aggravate, gender inequalities.

This study has provided a gender framework to the debates on tax equity in formalization processes. First, the study adds to the debate about *narrower or broader definitions of taxes in the informal sector*, suggesting that the broader version contributes to more egalitarian policymaking. As observed, women traders had to make flat payments on all fronts. A broad conceptualization that includes statutory taxes and tax‐like payments, and all the formal and informal actors involved, helps avoid underestimating the amount of tax and tax‐like payments borne by women that lead to an even greater taxation gender bias.

Second, the study advances understanding of the concept of *implicit gender biases* in public policies by showing evidence of harassment in tax collection and flat rates used to tax informal traders that earn differently—a new perspective to Stotsky's initial concept. What looked like gender‐neutral tax policies were, in reality, gender‐blind, as they did not address these discriminatory issues explicitly. The gender framework used to study the Nigerian case is relevant for marketplaces in other countries with large and feminized informal trade sectors coupled with gender‐blind tax policies.

The informal sector, and informal trading, are central to many country economies worldwide. In many low and lower‐middle‐income countries, these sectors are largely female. Overall, we concur with Lahey (2018) that better policy responses should support accurate calculations of profits, and that women need long‐term positive support to formalize their informal businesses, rather than strict policing and enforcement of presumptive taxation.

## CONFLICT OF INTEREST

The authors declare no conflict of interest.

## ENDNOTES

1 This article is based on a longer working paper submitted to the International Centre for Tax and Development (ICTD) at the Institute of Development Studies (IDS), United Kingdom, in 2019.

2 “Market traders” are vendors whose activity is circumscribed to marketplaces. This excludes sellers in open‐air locations such as sidewalks and transport junctions.

3 “Local markets” are spaces where goods are sold by small business traders. “Formal local markets” have a building, lockable gates, cement stalls, and are often located in rural and urban centers. “Informal local markets” have no constructed building or visible delimitations such as gates or fences but are popularly known as regular marketplaces. Informal markets are often located on the outskirts of rural and urban centers. Both are part of the informal sector and taxed through presumptive taxation.

4 However, each state has varying levels of poverty and infrastructure access that could affect market development and gender perceptions. Kaduna state also has many ethnic groups.

5 Besides official sources, we contacted the National Association of Nigerian Traders.

6 See https://taxsummaries.pwc.com/nigeria for an overview (accessed on October 30, 2021).

7 In Nigeria, “state taxes” refer to subnational taxes applying only in one of the 36 states of Nigeria, whereas “federal taxes” or “national taxes” apply to all the states forming the Federation of Nigeria.

8 However, for a future study we would seek to include market unions and associations as in all markets visited, they play a crucial role in tax collection.

9 The law also sets specific tax rates for slaughter stock, ranging from N500 to N2,000 (US$1.40–US$5.50). Assuming that the slaughter stock rates are in addition to the general presumptive tax rate (a point the tax law does not clarify), the maximum that any market trader should pay is N4,500 (US$12.40).

10 However, culture and tradition may still influence the gender of other services such as barbering and hairdressing.

11 “Unpaid care work” refers to social gender roles that denote women and girls as free providers of care work such as looking after children, older family members and the sick, preparing food, cleaning, and collecting water and fuel.

12 Taking products sold as the base on which to adjust segmented tax rates is a second‐best option given that women earn less than men even for the same types of products and services sold.
